# Burden of rotavirus gastroenteritis in the Middle Eastern and North African pediatric population

**DOI:** 10.1186/1471-2334-11-9

**Published:** 2011-01-07

**Authors:** Hanane Khoury, Isla Ogilvie, Antoine C El Khoury, Yinghui Duan, Mireille M Goetghebeur

**Affiliations:** 1 BioMedCom Consultants inc., 1405 TransCanada Highway, Suite 310, Montreal, Quebec, H9P 2V9, Canada; 2 Merck & Co, West Point, PA, 19486, USA; 3 Lehigh University, Bethlehem, PA, 18015, USA

## Abstract

**Background:**

Rotavirus gastroenteritis (RVGE) is the most common cause of severe childhood diarrhea worldwide. Objectives were to estimate the burden of RVGE among children less than five years old in the Middle East (Bahrain, Iran, Iraq, Israel, Jordan, Kuwait, Oman, Qatar, Saudi Arabia, Syria, UAE, Yemen), North Africa (Algeria, Egypt, Libya, Morocco, Tunisia) and Turkey.

**Methods:**

A comprehensive literature search was conducted in major databases on the epidemiology and burden of rotavirus among children less than five years old between 1999 and 2009. Data from each country was extracted and compared.

**Results:**

The search identified 43 studies. RVGE was identified in 16-61% of all cases of acute gastroenteritis, with a peak in the winter. RVGE-related hospitalization rates ranged from 14% to 45%, compared to 14%-28% for non-RVGE. Annually, RVGE caused up to 112 fatalities per 100,000 in certain countries in the region. Hospitalization costs ranged from $1.8 to $4.6 million annually, depending on the country. The most recent literature available showed that G1P[8] was the most prevalent genotype combination in 8 countries (range 23%-56%). G2P[4] was most prevalent in 4 countries (26%-48%). G9P[8] and G4P[8] were also frequently detected.

**Conclusions:**

RVGE is a common disease associated with significant morbidity, mortality, and economic burden. Given the variety and diverse rotavirus types in the region, use of a vaccine with broad and consistent serotype coverage would be important to help decrease the burden of RVGE in the Middle East and North Africa.

## Background

Rotavirus remains the most common cause of severe childhood diarrhea worldwide and of diarrheal mortality in developing countries [1]. The main symptoms of rotavirus gastroenteritis (RVGE) are fever, abdominal pain, lethargy, diarrhea and vomiting that may lead to hypovolemic shock and dehydration [2,3]. Severe cases may lead to death [4]. The World Health Organization (WHO) estimates that 527,000 children under the age of five years die of rotavirus disease each year [5]. Children in the poorest countries account for 82% of rotavirus deaths [6].

Rotavirus is transmitted by the fecal-oral route [2]. Infection rates for rotavirus are highest in children under five years of age, with 95% of children between the age of three and five years affected [7]. There is seasonality to rotavirus infection, with the majority of cases in temperate climates occurring in the winter months between November and February [2,8]. Seasonality in tropical and developing countries is less marked [7].

Three of the seven sero-groups of rotavirus identified affect humans, known as groups A-C. The most dominant, group A, causes diarrheal diseases worldwide [2]. Group A rotaviruses are classified into G and P-types, which are determined by the two outer layer viral proteins, VP7 and VP4, respectively. These two proteins elicit neutralizing antibody responses and therefore, protection from infection and disease is believed to be type-specific [9]. Rotaviruses are ubiquitous in the animal kingdom, and therefore, interspecies transmission and more importantly, exchange of genetic material between animal and human strains through re-assortment can lead to the emergence of novel rotavirus strains of epidemiological significance [9].

The incidence of infection with particular group A rotavirus serotypes and genotypes varies between geographical areas during a rotavirus season, and from one season to the next [10]. Globally, viruses carrying either G1, G2, G3, G4, G9 and P[4] or P[8] are the most common causes of rotavirus disease in humans. G12 is also recognized as an emergent serotype, that may become important in human disease [11].

Often, children suffering from RVGE require outpatient medical care, but in the presence of dehydration, admission to emergency or hospitalization and intravenous rehydration are necessary. Each year worldwide, rotavirus causes approximately 111 million episodes of gastroenteritis requiring only home care, 25 million office visits, and 2 million hospitalizations [6]. By the age of five years, nearly every child will have an episode of RVGE, one in five will visit a clinic, and one in 65 will be hospitalized [6]. Thus, RVGE imposes a heavy burden, not only by incurring direct medical costs, but also indirect costs due to productivity loss and other expenses [3,12,13]. Currently available rotavirus vaccines protected against severe RVGE and were well tolerated; the implementation of immunization programs would be expected to reduce disease burden [3].

Burden of illness data specific to the Middle East and North Africa is limited. The purpose of this study was to conduct a comprehensive literature review on the burden of rotavirus acute gastroenteritis on the pediatric population in these regions.

## Methods

### Literature search strategy

To identify and retrieve articles pertaining to the impact of rotavirus infection on the pediatric population (≤5 years, unless otherwise specified) in the Middle East and North Africa, a comprehensive literature search was conducted in the National Library of Medicine's Pubmed, the Center for Disease Control (CDC) rotavirus global surveillance (http://www.cdc.gov/rotavirus/global_surveillance/surveillance.htm), and the WHO (http://www.who.int/nuvi/rotavirus/en/). The search, limited to articles published from 1999 to 2009, covered the Middle East (Bahrain, Iran, Iraq, Israel Jordan, Kuwait, Oman, Qatar, Saudi Arabia, Syria, UAE, Yemen), North Africa (Algeria, Egypt, Libya, Morocco, Tunisia), as well as Turkey for its regional proximity. Search terms included: rotavirus, outcome*, mortality, death, incidence, prevalence, serotype, strain, cost*, economic*, burden, and resource use. Reviews and case studies were excluded.

### Data extraction and analysis

For all studies, dates reported for data presented refer to the date when studies were conducted, which was often several years before the publication date.

In the case where several surveillance studies are published for a single country, a pooled average of the proportion of RVGE among cases of acute gastroenteritis was calculated and reported. Ranges across studies were also reported for each country. Where available, data on infection seasonality was collected and reported, in addition to variation over time in the proportion of RVGE.

Data was extracted for each serotype. Figures for distribution of rotavirus genotype combinations were taken from the most recent available data, except for Turkey. For this country, data from a prospective survey from 2004-2005 [14] was used to replace a more recent publication (2005-2006 Ceyhan study [15]), due to discrepancies in the serotype data reporting (combined serotyped data in the Ceyhan study [15] adds up to 113%). Where two studies from the same year and the same country showed a similar serotype distribution, a weighted average across the studies was calculated to present as one figure. All other figures were reported as originally described in the source documents.

Mortality data included annual fatalities and mortality rates per 100,000 population under five years of age. For health outcomes of rotavirus acute gastroenteritis, data was extracted on disease severity as measured by the 20-point Vesikari scoring system [16], and the severity and proportion of patients suffering from dehydration due to rotavirus acute gastroenteritis. The Vesikari scale is a numerical system used to assess RVGE disease severity, based on the duration and intensity of diarrhea and vomiting, intensity of fever and dehydration, and the need for treatment and hospitalization [16]. A Vesikari score ≥11 is indicative of severe disease.

For healthcare resource use data, the following parameters were extracted for comparison across countries: hospital admission rates, need for intravenous rehydration, and duration of hospital stay. Cost-of-illness data included direct medical costs, out-of-pocket expenditures, and indirect costs attributed to lost productivity by parents of children suffering from RVGE. Costs are reported in 2008 US dollars.

No statistical analyses were performed for this review.

## Results

### Studies included in this review

**Figure 1:**
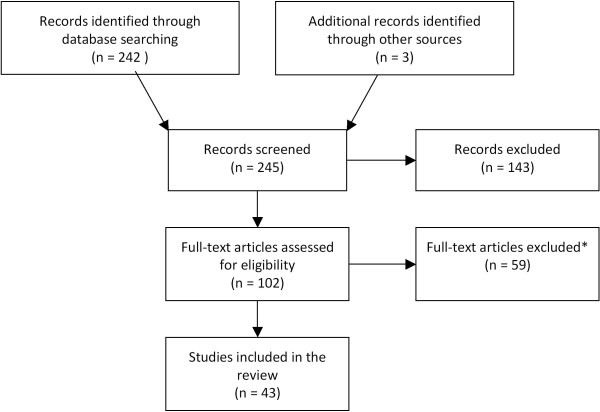
Flow chart representing the selection of relevant citations

As shown in Figure 1, this literature search recovered 43 citations which contain relevant data pertaining to acute gastroenteritis associated with rotavirus infection on the following topics: incidence and proportion of RVGE among cases of acute gastroenteritis (n = 37 studies), serotype distribution (n = 25), mortality (n = 1), disease severity and outcomes (n = 9), healthcare resource use (n = 11), and costs (n = 2). A summary of data sources by country is presented in Table 1.

**Table 1:** Literature capture and data sources by country

Country	Epidemiology			Genotype combination data	Morbidity and Mortality		Disease Burden	
			
	Incidence	Proportion RVGE	Seasonality		Disease severity	Mortality	Resource use	Costs
Multicountry*		[18]^†^				[55]^‡^		
Algeria						[55]		
Bahrain						[55]		
Egypt	[21]	[17-21]	[19,21]	[17,17,18,18,21,53]	[19,21,51]	[55]	[19,51]	
Iran		[18,22-27]	[22,23,26,27]	[18,22,26]		[55]	[25]	
Iraq		[18,28]		[28]		[55]		
Israel				[52]		[55]	[52,57]	[52]
Jordan		[18,29]		[18]	[29]	[55]	[29]	
Kuwait		[30]		[30]		[55]		
Libya		[18,31,32]	[31]	[32]		[55]		
Morocco		[18,33]	[33]	[18,33]	[33]	[55]	[33]	
Oman		[18,34,35]	[34]	[18,34,35]		[55]	[34]	[34]
Qatar						[55]		
Saudi Arabia		[36-40]	[37]	[36-38]		[55]		
Syria		[18]				[55]		
Tunisia		[18,41-46]	[42,45,46]	[41,44,44,46]	[42]	[55]		
Turkey		[14,15,47-50]	[14,15,49,50]	[14,15,48]	[15,47,50]	[55]	[15,47,50]	
UAE						[55]		
Yemen		[18]		[18]		[55]		

*Where country-specific data was available within a multicountry study, it has been extracted. Therefore, the relevant reference has been added to individual countries above

†Egypt, Iran, Iraq, Jordan, Libya, Morocco, Oman, Syria, Tunisia, and Yemen

‡All countries

### Epidemiology of rotavirus acute gastroenteritis

Data on the proportion of RVGE was available from the following countries: Egypt [17-21], Iran [18,22-27], Iraq [18,28], Jordan [18,29], Kuwait [30], Libya [18,31,32], Morocco [18,33], Oman [18,34,35], Saudi Arabia [36-40], Syria [18], Tunisia [18,41-46], Turkey [14,15,47-50], and Yemen [18]. No studies were found from Algeria, Bahrain, Israel, Qatar, and the United Arab Emirates. Most studies contained data on the proportion of RVGE rather than RVGE incidence. Only one study contained incidence data [21].

#### Incidence

In Egypt, a population-based cohort study of children under three years of age reported age-related incidence of 0.61 rotavirus diarrheal episodes per person-year between 1995 and 1996 [21]. In this cohort, age-related incidence was highest in children aged 6 to 11 months.

#### Proportion and seasonality of RVGE

**Figure 2:**
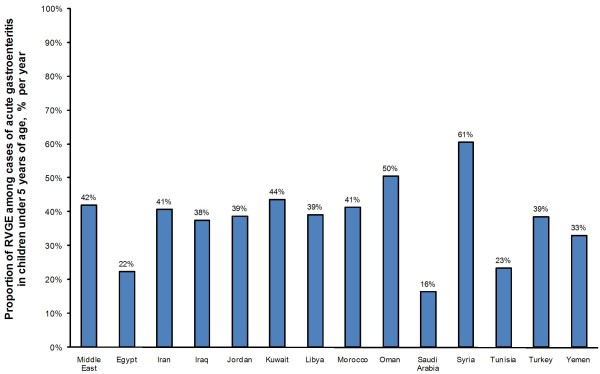
Mean overall proportion of rotavirus gastroenteritis (% of reported acute gastroenteritis cases) by country

When looking at the most recent studies that report data on the proportion of RVGE for children under five years of age in Middle Eastern and North African countries, the proportion of RVGE among cases of acute gastroenteritis ranged from 16% to 61% per year (Figure 2). Among the countries with the lowest proportion of RVGE (16% to 23%) were Saudi Arabia [36-40], Tunisia [18,41-46], and Egypt [17-19,51]. Those with the highest proportion of RVGE included Syria (61%) [18], Oman (50%) [18,34,35], and Kuwait (44%) [30]. One study covered the WHO Eastern Mediterranean region as a whole, including many of the above countries (Egypt, Iran, Iraq, Jordan, Libya, Morocco, Oman, Syria, Tunisia, and Yemen) as well as Afghanistan and Sudan [18]. In this study, the overall annual prevalence of RVGE among reported episodes of gastroenteritis in children under five years of age was 42% [18].

A number of countries reported seasonality data including Egypt [19,21], Iran [22,23,26,27], Libya [31], Morocco [33], Oman [34], Saudi Arabia [37], Tunisia [42,45,46], and Turkey [14,15,49,50]. For most of these countries, the peak season for rotaviral gastroenteritis is in the winter from November to April. The exception to this is Egypt where rotaviral infection peaks in July to November [19,21].

#### Variation in the proportion of RVGE over time

**Figure 3:**
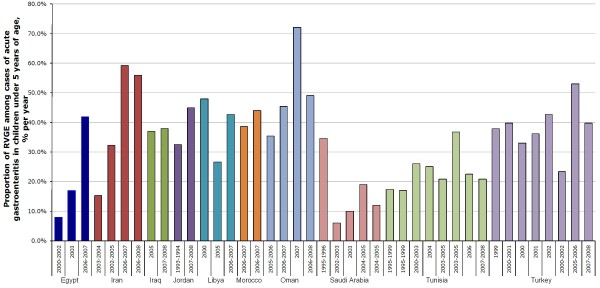
**Proportion of rotavirus gastroenteritis (% of reported acute gastroenteritis cases) by country and year of study**. Dates refer to when studies were conducted.

As illustrated in Figure 3, the proportion of RVGE among acute gastroenteritis cases appears to have increased over time in Egypt (from 8% to 42% between 2000 and 2007) [18,19] and Iran (from 15% in 2003-2004 to 59% in 2006-2007) [22,27]. Proportion of RVGE in Saudi Arabia has fallen from 35% in 1995-1996 to around 12% since 2002-2003 [38,40]. Proportion of RVGE in the other countries for which data over time was available has remained relatively stable.

### Rotavirus genotype combinations in the Middle East and North Africa

#### Distribution of genotype combinations

**Figure 4:**
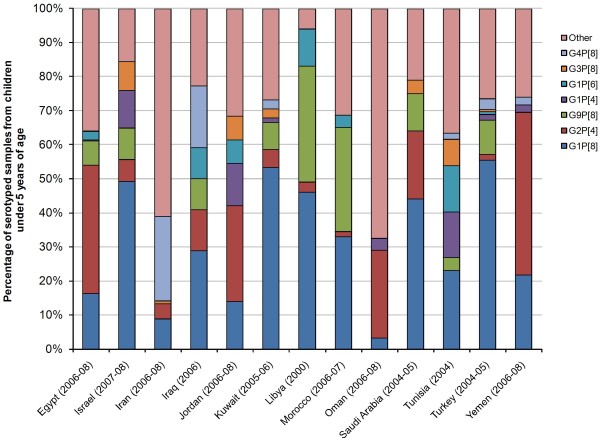
**Distribution of rotaviral genotype combinations in the Middle East and North Africa by country, most recent literature available**. NB: "Other" includes the proportion of rare and mixed genotype combinations, and non- or partially-typable serotypes.

As shown in Figure 4, G1P[8] was the most prevalent genotype combination in eight of the 14 countries for which recent data was available (Israel [52], Iraq [28], Kuwait [30], Libya [32], Morocco [33], Saudi Arabia [37], Tunisia [44], and Turkey [14]), with a proportion ranging from 23% to 56% of all genotyped samples. G2P[4] was the most prevalent genotype combination in four countries (Egypt [17,18], Jordan [18], Oman [18,34], and Yemen [18]; range 26%-48%), and was detected in every country except Tunisia. G9P[8] was detected in 10 of the 14 countries for which there was literature available (Egypt [17,18,53], Israel [52], Iraq [28], Kuwait [30], Libya [32], Morocco [18,33], Oman [35], Saudi Arabia [36-38], Tunisia [41,44], and Turkey [14,15,48]), and was highly prevalent in Morocco [33] and Libya [32] (31% and 34% of genotype combinations, respectively). G4P[8] was most prevalent in Iran [18] (27% of genotyped samples, respectively), and was present in Iraq [28], Kuwait [30], Tunisia [44,46], Turkey [14], and Yemen [18] (proportion range 2%-18%).

Other genotype combinations were also present in multiple countries as follows. G1P[4] (Egypt, Israel, Jordan, Kuwait, Oman, Tunisia, Turkey and Yemen; proportion range 0.2%-14%); G1P[6] (Egypt, Iraq, Jordan, Libya, Morocco, Pakistan, Turkey, and Tunisia; range 1%-14%); G3P[8] (Israel, Iran, Jordan, Kuwait, Saudi Arabia and Tunisia). Rarer genotype combinations, each present in six countries or fewer, accounted for 10% or less of the total genotype combinations (Table 2). Mixed genotype combinations, including G1G2 and G1G4, were detected in most countries (2%-25% of the total rotavirus positive samples). Non-typable and partially typed serotypes accounted for a significant proportion of serotype samples, ranging between 5% and 20% for non-typable, and 5% to 19% for partially typable samples.

**Table 2:** Rare genotype combinations from the Middle East and North Africa by country (recent studies)

	Egypt	Israel	Iran	Iraq	Jordan	Kuwait	Morocco	Oman	Saudi Arabia	Tunisia	Turkey	Yemen	
		
Serotype	2006-08	2007-08	2006-08	2006	2006-08	2005-06	2006-07	2006-08	2004-05	2004	2004-05	2006-08	n of countries
**G2P[8]**	5.96%	2.80%	--	--	--	1.30%	0.70%	--	--	--	1.60%	2.20%	6
**G4P[6]**	--	--	--	1.50%	--	1.30%	--	--	--	3.80%	--	--	4
**G2P[6]**	1.88%	--	--	--	1.80%	--	9%	--	--	--	--	--	3
**G3P[4]**	--	7.40%	--		--	--	--	--	--	3.80%	1.60%	--	3
**G12P[8]**	--	4.60%	--		--	--	--	--	4%	1.90%		--	3
**G9P[4]**	--	0.90%	--		--	--	--	2.9%	--	--	1.60%	--	3
**G9P[6]**	--	--	--	1.50%	--	--	--	--	--	--	3.10%	--	2
**G12P[6]**	6.96%	--	--		--	--	--	--	--	--	--	--	1
**G3P[9]**	--	--	0.78%		--	--	--	--	--	--	--	--	1
**G2P[10]**	--	--	--	--	--	--	--	1.3%	--	--	--	--	1
**G1P[10]**	--	--	--	--	--	--	--	1.2%	--	--	--	--	1
**G3P[6]**	--	--	--	--	--	--	--	--	--	1.90%	--	--	1
**G4P[4]**	--	--	--	--	--	--	--	--	--	1.90%	--	--	1
**G8P[4]**	--	--	--	--	--	--	--	--	--	--	1.50%	--	1
**G1P[11]**	--	--	--	--	--	--	--	--	--	--	--	2.20%	1
**G9P[11]**	--	--	--	--	--	--	--	--	--	--	--	2.20%	1

#### Evolution of genotype combinations over time

**Figure 5:**
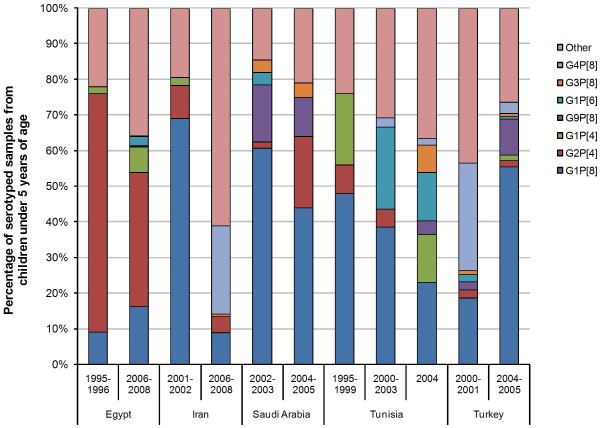
**Distribution of rotaviral genotype combinations in the Middle East and North Africa over time, from recent literature**. NB: "Other" includes the proportion of rare and mixed genotype combinations, and non- or partially typable serotypes.

Studies from six countries reported distribution over time of fully genotyped samples from children ≤5 years of age: Egypt [17,18,21], Iran [18,22,26], Oman [18,34,35], Saudi Arabia [36,38], Tunisia [44], and Turkey [14,48] (Figure 5). In Egypt, the proportion of G2P[4] fell, and G1P[8] increased between 1995-1996 and 2006-2008 [17,18,21]. Over the same time frame, G9P[8] was detected (7%). In Iran, G1P[8] proportion declined, and G4P[8] became the most prevalent genotype combination in 2006-2008 [18,22,26]. In Saudi Arabia, there was a decrease in both G1P[8] and G9P[8] (16% to 11%), while G2P[4] increased from 2002-2003 to 2004-2005 [36,38]. In Tunisia, G1P[8] and G1P[6] were replaced by a variety of genotype combinations including G1P[4], G9P[8], and G3P[8] [44]. Finally in Turkey, the predominant genotype combination G1P[8] increased in proportion, while G4P[8] declined [14,15,48].

Two studies, both from Tunisia, covered the emergence of new serotypes in North Africa [41,46]. The earlier study by Trabelsi et al, described the emergence of a reassorted strain G1P[4] in the Sousse region in the 1998-1999 season. G1, G2, and G4 strains were prevalent at this time, with no detection of G3 or G9 [46]. The second, a prospective study of five hospitals in Eastern-Central Tunisia described the emergence of G9 serotypes during 2003-2005, further characterized as G9P[8] [41].

### Morbidity and mortality due to rotavirus acute gastroenteritis

#### Disease severity

Data pertaining to disease severity and dehydration due to RVGE in the Middle East and North Africa was available from the following countries only: Egypt [19,21,51], Jordan [29], Morocco [33], Tunisia [42], and Turkey [15,47,50]. Dehydration was a common health outcome reported by all studies, affecting around 50% of children with RVGE [19,21,42,50,51]. In 3% to 25% of cases, dehydration was classified as severe [15,19,21,42,54]. Furthermore, disease severity, measured on the Vesikari scale, was explored by one study from Morocco [33] and three Turkish [15,47,50] studies. In Morocco, a prospective surveillance study of 345 hospitalized children with rotavirus estimated a median Vesikari score of 14 [33]; this score was significantly higher than the one obtained among children with non-RVGE (*P *= 0.03). In Turkey, prospective hospital-based studies showed that between 37% and 90% of children with rotavirus had severe disease on the Vesikari scale [15,47,50].

#### Mortality

**Figure 6:**
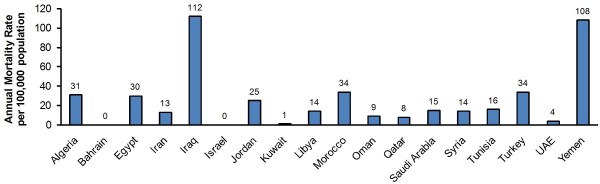
Mortality due to rotavirus in the Middle East and North Africa by country

Information on mortality due to RVGE in the Middle East and North Africa was limited to the WHO report on death estimates from all countries of interest [55] In the countries covered in this study, fatalities due to RVGE ranged from a low of <10 per year (Bahrain, Israel, Kuwait, and Qatar) to a high of 4,723 per year (Iraq). This translates into annual mortality rates of 0 to 112 per 100,000 infants under five years of age (Figure 6) [55]. When the overall pediatric population below five years of age was considered, the average mortality rate from the 20 countries was estimated at 39 per 100,000 per year (17,766 rotavirus deaths per 45,437,000 children <5 years of age--estimate based on demographic indicators from UNICEF [56]).

### Healthcare resource use

Overall, 11 studies reported healthcare resource use data from Egypt [19,51], Iran [25], Israel [52,57], Jordan [29], Morocco [33], Oman [34], and Turkey [15,47,50].

#### Hospital admissions

In Egypt, two hospital-based studies reported hospital admission rates due to RVGE among young children. This rate varied between 14% among children presenting with diarrhea at a hospital in Southern Egypt [51], and 45% among those presenting at two government referral hospitals in the Nile River Delta [19]. Hospitalization rates were 39% among toddlers with RVGE (<2 years) in Iran [25], and 31% among children younger than five years of age in Turkey [50]. Interestingly, the Turkish prospective survey, involving 920 patients admitted for acute gastroenteritis, found that rotavirus-positive gastroenteritis caused a significantly higher rate of hospital admissions versus non-RVGE (31% vs 14% non-RVGE; *P *< 0.01) [50]. In Oman, a prospective hospital-based surveillance study estimated that, by age five years, one in 16 children will require hospitalization due to rotavirus [34].

#### Intravenous rehydration

At least 90% of hospitalized children with RVGE required intravenous rehydration in Morocco [33], Oman [34], and Turkey [15,47]. This figure was 63% in a prospective survey from Jordan [29], and 67% among children aged <18 months in Israel [57].

#### Duration of hospital stay

Data from three countries indicated that RVGE among young children required hospitalization of at least three days, as reported in a prospective surveillance study from Oman (median: 3 days) [34], and a population-based cohort study in Israel (mean: 3.0 ± 2.9 days) [52]. In Turkey, the mean hospital duration was longer for RVGE than non-rotavirus disease (5.5 ± 5.1 days vs 3.3 ± 3.1 days non-rotavirus) [50]. A prospective study of three pediatric departments in Israel reported that hospital stay was longer among younger children with nosocomial RVGE (3.5 vs 2.1 days for children <6 months old and those >26-48 months, respectively) [57]. In this study, hospital duration was 3.5 and 2.1 days for children <6 months and those between 36 and 48 months, respectively [57].

### Economic burden associated with RVGE

Cost of illness data was limited to studies from Israel [52] and Oman [34]. In Israel, the total cost of hospitalization (direct medical costs) was estimated at 2008 US $1,117 per patient for an average of three hospital days, for a total of $4.59 million per year for all Israel patients [52]. One episode of RVGE was estimated to incur out-of-pocket expenses of $281, for a national annual estimate of $1.15 million paid by parents (extra diapers, transportation, over-the-counter medications, special diet, medical consultations), and caused productivity loss of four to six days of work at a cost of $476 per patient hospitalized, for a national annual estimate of $1.95 million for indirect costs [52]. In Oman, Al Awaidy et al (2009) [34] estimated the direct cost for three days of hospitalization at $539 per event, or $1.80 million per year. Out-of-pocket and indirect costs were not reported in this study.

## Discussion

Based on data collected from 44 studies in the Middle East and North Africa, this analysis shows that rotavirus imposes a heavy burden among children less than five years of age. Overall, the annual proportion of RVGE among reported episodes of pediatric gastroenteritis in the Middle East and North Africa region was 42% [18]. This figure is similar to a published estimate of the proportion of RVGE (43%) from a prospective multicountry study in Western Europe [58], further emphasizing the ubiquitous nature of the disease [7]. However, when Middle Eastern and North African countries were compared to each other, large variations in proportion of RVGE estimates were observed, with a low of 16%-23% reported in Saudi Arabia, Tunisia, and Egypt, and a high of 44%-61% in Syria, Oman, and Kuwait. These variations may reflect actual differences in RVGE proportion but may also be related to variations in study design, which limit comparability across countries.

A considerable amount of information on serotype distribution in the countries of interest was retrieved, highlighting the predominance of G1P[8] and G2P[4]. These genotype combinations are also predominant in Western Europe [59]. In the present study, the proportion of non-typable and partially typable genotype combinations varied widely across countries [22,26,36,37]. The reason for this discrepancy is not clear, although it might be due to differences in study design and setting, or to laboratory practice differences from country to country.

Several studies assessed the evolution of serotype distribution over time and the emergence of new rotavirus strains in the Middle East and North Africa. All of these prevalent (G2P[4] [36,38], G4P[8] [18,22,26], G3P[8] [44]) and emerging (G9P[8] [17,18,21,41]; G1P[4] [46]) serotypes belong to the most commonly described strains of rotavirus that are responsible for gastroenteritis disease in humans [10]. Interestingly, G12, a recently emerging serotype detected in Europe, Asia, and the Americas [11,60,61], has not been reported in any of the studies captured in this review.

A wide inter-country variation was noticed in mortality rates due to RVGE, with the highest rates reported in Iraq and Yemen compared to less than one fatality in Bahrain, Israel, and Kuwait (Figure 6). Based on demographic indicators from UNICEF, the average annual mortality rate in the region was estimated at 39 per 100,000. This rate is considerably higher than in Europe, where rotavirus rarely results in child death (mortality rate below 10 per 100,000) [55]. These inter-country and inter-region variations are in agreement with previous reports showing that children in the poorest countries account for 82% of rotavirus deaths [6]. Because of data limitations, it cannot be concluded from this study whether differences in mortality rates are due to variations in clinical management of the disease.

The hospital admission rates and the use of intravenous rehydration were similar to European figures reported in the multicentre RVGE Epidemiology and Viral types in Europe Accounting for Losses in public health and society (REVEAL) study [62]. As well, the higher rates of hospitalization and higher disease severity for rotavirus versus non-rotavirus acute gastroenteritis reported in this review were in line with data reported in Western Europe [63].

Rotavirus cost information was very limited in the Middle East and North Africa. Per episode of RVGE, direct medical costs in the Middle East and North Africa ($467 to $1,117) were lower than those reported in Western Europe (ranging from 2008 US $1,949 in the UK to $2,398 in Sweden) [62].

Study limitations are worth mentioning. Most published articles retrieved in this study reported serotype and epidemiological data; information on the burden of RVGE in terms of mortality, morbidity, and economic burden was limited. This in term restricted the evaluation of the global burden for the region. Most recent available data was considered to describe and compare serotype distribution across countries; however, the only available data was not necessarily recent and did not correspond to the same time frame in all countries. For example, the only serotype information for Libya was published in 2000 [32], therefore conclusions on serotype distribution for this country may have changed, and comparison with 2008 data from other countries would be limited. Moreover, due to data limitations, a clear relationship between certain rotavirus genotypes and disease severity could not be established. Finally, variations in study setting and design may affect comparability of data.

## Conclusions

RVGE is a common disease associated with significant morbidity, mortality and costs in the Middle East and North Africa. The results of this study may be useful as background information to the planning and implementation of efficient vaccination programs. Given the variety and diverse rotavirus types in the region, a vaccine with broad and consistent serotype coverage would be important to help decrease the burden of RVGE in the Middle East and North Africa.

## Competing interests

HK, IO, and MMG declare that they have no competing interests. AEK is an employee of Merck Sharp & Dohme Corp. and potentially owns stock and/or holds stock options in the Company. YD is a fellow at Lehigh University; her fellowship was funded by Merck & Co.

## Authors' contributions

HK & IO developed the search algorithm and drafted the manuscript. AEK, YD, and MMG participated in the design of the methodology and drafting of the manuscript. All authors read and approved the final manuscript.

## Pre-publication history

The pre-publication history for this paper can be accessed here:

http://www.biomedcentral.com/1471-2334/11/9/prepub
